# The London Commercial Travellers and the Hospital Sunday Fund

**Published:** 1904-12-31

**Authors:** 


### THE LONDON COMMERCIAL TRAVELLERS AND THE HOSPITAL SUNDAY FUND.

On the 17th inst., on the invitation of the Lord Mayor, the London Commercial Travellers met together at the Mansion House to consider the best means of increasing the collections on Hospital Sunday to £100,000. The Lord Mayor was supported by Sir E. Hay Currie, Archdeacon Sinclair, Sir Frederick Treves, Sir Joseph Dimsdale, and Mr. C. A. Body.

The Lord Mayor said that the commercial travellers had special opportunities of introducing the claims of the Hospital Sunday Fund to the notice of their fellow-citizens, and he believed that they could improve these opportunities without prejudice to their own charities, which they supported so well.

Sir Joseph Dimsdale proposed that the hospitals of London were worthy of support, especially at the present time. He said that the hospitals of the metropolis were really Imperial institutions, and he asked those present as the busy bees of the metropolis to ask those whom they met to support the hospitals as charities which, regardless of creed or nationality, gave comfort and support to the sick and alleviated suffering.

Sir Frederick Treves, in seconding the resolution, said that owing to the advance of medicine and surgery, there were now innumerable methods of treatment requiring special appliances and antiseptic conditions which were hardly attainable in private houses, and the work of the hospitals therefore played a more important part than formerly, and was directly beneficial to different classes of the population. The London hospitals, he said, were metropolitan only in their locality. Patients were to be found in every London hospital from every part of the country and from practically every colony under the British flag.

Mr. J. E. Alger, on behalf of the meeting, thanked the Lord Mayor for inviting the commercial travellers, and assured him of their desire that the movement should have a practical result. They were sensible of the benefits and of the necessities of the hospitals and would do their best to bring to a successful issue the effort they were called upon to make.

The resolution was then carried unanimously.

Mr. Charles A. Body moved, and Mr. J. Eaton seconded, the following resolution:—"That this meeting of London commercial travellers, being desirous of assisting the hospitals of London, pledge themselves to do their utmost to raise a fund to be added to the collection at St. Paul's Cathedral on next Hospital Sunday, June 25, 1905." It was explained that the object of adding the money to the St. Paul's collection was to gain the grant which Mr. Herring was good enough to add. That meeting was not asked to subscribe any money, but specially prepared collecting cards would be sent to all who had been invited.

The resolution was unanimously adopted, and on the motion of Mr. H. S. Windsor and Mr. A. J. Brett, a vote of thanks was given to the Lord Mayor.

